# Absence of Nkx2-3 induces ectopic lymphatic endothelial differentiation associated with impaired extramedullary stress hematopoiesis in the spleen

**DOI:** 10.3389/fcell.2023.1170389

**Published:** 2023-04-05

**Authors:** Fanni Gábris, Gabriella Kiss, Balázs Szirmay, Árpád Szomor, Gergely Berta, Zoltán Jakus, Zoltán Kellermayer, Péter Balogh

**Affiliations:** ^1^ Department of Immunology and Biotechnology, University of Pécs Medical School, Pécs, Hungary; ^2^ Lymphoid Organogenesis Research Group, Szentágothai Research Center, University of Pécs, Pécs, Hungary; ^3^ Department of Laboratory Medicine, University of Pécs Medical School, Pécs, Hungary; ^4^ Department of Internal Medicine, University of Pécs Medical School, Pécs, Hungary; ^5^ Department of Medical Biology and Central Electron Microscope Laboratory, Medical School, University of Pécs, Pécs, Hungary; ^6^ Department of Physiology, Semmelweis University, Budapest, Hungary

**Keywords:** NKX2-3, spleen, lymphatic endothelium, Prox1, hematopoiesis, stroma

## Abstract

The red and white pulps as two main parts of the spleen are arranged around distinct types of vasculature, and perform significantly different functions in both humans and mice. Previous observations indicated a profound alteration of the local vessel specialization in mice lacking Nkx2-3 homeodomain transcription factor, including contradictory results suggesting presence of an ectopic lymphatic vascular structure. Furthermore, how the absence of Nkx2-3 and the consequential changes in endothelial components affect the extramedullary hematopoietic activity restricted to the splenic red pulp is unknown. In this work, we investigated the role of Nkx2-3 homeodomain transcription factor as a major morphogenic determinant for vascular specification, and its effect in the extramedullary hematopoiesis following acute blood loss and pharmacological stimulation of megakaryocyte differentiation after treatment with thrombopoietin-receptor mimetic Romiplostim. We found that, in mice lacking Nkx2-3, Prox1-positive lymphatic capillaries containing gp38/CD31 double positive lymphatic endothelial cells develop, arranged into an extensive meshwork, while the Clever1-positive venous segments of red pulp blood vasculature are absent. This lymphatic endothelial shift is coupled with a severely compromised splenic erythropoiesis and a significantly reduced splenic megakaryocyte colony formation following Romiplostim treatment in mice lacking Nkx2-3. These findings indicate that the shift of microvascular patterning in the absence of Nkx2-3 includes the emergence of ectopic Prox1-positive lymphatic vessels, and that this pivoting towards lymph node-like vascular patterning is associated with an impaired reserve hematopoietic capacity of the splenic red pulp.

## 1 Introduction

The spleen is the largest single peripheral lymphoid organ with diverse biological functions in both humans and mice (reviewed in (Mebius and Kraal, 2005; Kashimura, 2020; Crane et al., 2021)). In addition to performing various immune reactions against blood-borne pathogens, it is also involved in organismal iron homeostasis, removal of effete red blood cells and stress-erythropoiesis following acute blood loss. After birth, the extramedullary hematopoietic activity of the spleen is largely quiescent, replaced by its main immunological functions to assist in B-cell development and homeostasis, and mount immune responses. The residual hematopoiesis is restricted to the red pulp (RP), whereas the splenic immune reactions involve both the white and red pulp.

These two distinct structural and functional compartments of the spleen are arranged around their specialized mesenchymal scaffolding, including their vasculature, and evolve during the prenatal and early postnatal period (Golub et al., 2018; Mende and Laurenti, 2021). The bulk segregation of the splenic RP (with regressing hematopoietic activity) and white pulp (with the emergence of immunological capacity, including the establishment of lymphoid stromal scaffolding) in mice occurs in the early postnatal period.

The preservation of the postnatal myelopoietic capacity of the spleen is linked to the presence of a specialized stromal subset and the stromal expression of Tlx1 located in the RP (Lenti et al., 2016; Oda et al., 2018; Lim and O'Neill, 2019). Tlx1 (formerly HOX11) controls the splenic development through a retinoic acid (RA)-dependent mechanism, whereas stromal cells in the RP *via* transcription factor WT1 (also promoting spleen organogenesis) influence the homeostasis of RP macrophages through CSF1 cytokine and CCL1 and CCL7 chemokines (Herzer et al., 1999; Bellomo et al., 2020). In addition, the RP vasculature has also been demonstrated to contain specific niches for the hematopoietic stem cells to supplement bone marrow blood cell development (Kiel et al., 2005; Inra et al., 2015).

In this postnatal splenic stromal specification, Nkx2-3 transcription factor has been identified as a key element, regulating both the vascular patterning of the RP as well as the structure of the marginal zone (MZ), two tissue-specific compartments of the spleen. Lack of Nkx2-3 in mice results in hypoplastic spleen, mainly due to the reduced RP and missing MZ including MAdCAM-1-positive marginal reticular cells (MRCs) and MZ macrophages (Pabst et al., 1999; Wang et al., 2000). In addition, in Nkx2-3 KO mice the splenic RP vasculature contains ectopic high endothelial venules (HEVs) mediating L-selectin dependent homing (Balogh et al., 2007; Czömpöly et al., 2011). Furthermore, LYVE-1^+^ dilated sac-like structures have also been identified in the Nkx2-3 null-mutant spleens, raising possible lymphatic endothelial cell (LEC) derivation, unlike to normal spleen where LYVE-1 expression is restricted to RP-associated megakaryocytes and platelets. These sac- or tube-like structures emerge in the early postnatal period in mutant mice in a process independent of lymphotoxin β-receptor (LTβR), while the exact cellular origin of these LYVE-1-positive structures in Nkx2-3-deficient mice has remained undetermined (Kellermayer et al., 2011). Importantly, the spleen in mice is largely devoid of lymphatic vasculature with clearly defined functionality, in contrast to lymph nodes or mucosal lymphoid tissues.

Although the spleen as a separate organ is allowed to form in the absence of Nkx2-3, its complex alteration in Nkx2-3-deficient mice raises the question of the extent of vascular alterations, and whether these stromal alterations extend beyond the arrangement of blood vessels. In this work, we investigated the possible LEC derivation of LYVE-1-positive structures, and whether the defective splenic architecture in the absence of Nkx2-3 affects the spleen’s capacity to activate extramedullary hematopoiesis in forced erythropoiesis or megakaryocyte differentiation. Our findings demonstrate that the lack of Nkx2-3 promotes the formation of Prox1-positive LEC capillaries, associated with the profound inhibition of the splenic stress hematopoiesis, thus extending the spectrum of mesenchymal cell functions affected by Nkx2-3, beyond defining the tissue-specific vasculature of the spleen. These findings identify Nkx2-3 as a crucial transcription factor defining the blood endothelial/LEC commitment in a tissue specific fashion, which, in turn, determines the extramedullary hematopoietic capacity of spleen.

## 2 Materials and methods

### 2.1 Experimental animals

Young adult (8–10 weeks old) Nkx2-3^−/−^ and wild-type female mice were obtained from our departmental SPF unit, and maintained on a BALB/c genetic background under minimal disease conditions. Nkx2-3 mice were bred in heterozygote-homozygote pairing and genotyped as described (Czömpöly et al., 2011). For inducing stress erythropoiesis, BALB/c and Nkx2-3^−/−^ mice were placed in a 50 ml restraining tube, followed by venipuncture of the lateral tail vein with a 26G needle. Approximately 200 µL blood was collected, which was repeated twice two days apart. Mice were sacrificed on the seventh day and their blood was collected for cellular and serological tests, while the bone marrow and spleen were processed for flow cytometric and immunohistological analyses. For stimulating megakaryocytes, BALB/c and Nkx2-3^−/−^ mice received Romiplostim intravenously (Nplate, Amgen Europe B.V.) at 300 ng/g body weight in a single dose in 100 µL volume diluted in PBS (Slayton et al., 2002; Sparger et al., 2018), after which the spleens were collected on the seventh day. Prox1-GFP bacterial artificial chromosome (BAC) lymphatic reporter–transgenic mice (Choi et al., 2011) were maintained in heterozygous form and were used for breeding (Prox1-GFP C57BL/6J crossed with BALB/c Nkx2-3^−/−^ to F2 generation) to obtain Prox1GFP-Nkx2-3−/− mice for lymphatic endothelial cell (LEC) visualization. Spleen samples from Stab1^−/−^ mice were provided by Dr. Sirpa Jalkanen, University of Turku, Finland (Karikoski et al., 2014). Mouse experiments were carried out in the animal facility of the Department of Immunology and Biotechnology, University of Pécs, under license numbers BA02/2000-16/2015 and BA02/2000-43/2021, with approval for the use of genetically modified organisms under license number SF/27-1/2014 issued by the Ministry of Rural Development, Hungary, in accordance with the guidelines set out by the Ethics Committee on Animal Experimentation of the University of Pécs, Hungary.

### 2.2 Antibodies and reagents

Rat monoclonal antibodies against mouse Ly76 antigen (clone TER-119), PerCP-Cy5.5-conjugated anti-mouse CD45 (clone 30F11), APC-conjugated anti-mouse CD31 (clone MEC13.3), and PE-labeled hamster anti-mouse podoplanin/gp38 mAbs (clone 8.1.1.) were obtained from BD Biosciences (Diagon Ltd., Budapest, Hungary). Anti-mouse Clever1 antibody [clone 9-11 (Palani et al., 2011)] was kindly provided by Dr. Sirpa Jalkanen, University of Turku, Finland. Biotinylated anti-mouse CD29 mAb (clone HMβ1-1) and anti-mouse CD41 (clone MWReg30) were purchased from BioLegend (Biomedica Hungaria, Budapest, Hungary). Rat mAb against mouse CD45 (clone IBL-3/16) conjugated with Alexa Fluor 647, FITC-conjugated rat mAb against mouse CR1/2 (clone #7G6) and rat mAb IBL-9/2 against red pulp endothelium (Balogh et al., 2007) were generated in our lab. The rat hybridoma clone M/K-2.7 against mouse VCAM-1 was obtained from ATCC (ATCC CRL-1909) and was used to produce hybridoma supernatant. FITC-conjugated goat anti-rat IgG and PE-streptavidin conjugates were purchased from BD Pharmingen (Diagon Ltd., Budapest, Hungary), Cy3-conjugated goat anti-rat IgG was purchased from BioLegend (Biomedica Hungaria, Budapest, Hungary). For immunohistochemistry we used ImmPRESS goat anti-rat IgG-HRP polymeric conjugate (Vector Laboratories, BioMarker, Gödöllő, Hungary). Other chemicals for buffers and histochemical substrates were purchased from Sigma-Aldrich.

### 2.3 Flow cytometry

After sacrificing the animals, their spleens were removed and the bone marrows were extracted by rinsing the femoral bone cavity with PBS using a 22G needle affixed to a 2 ml syringe. After dissociation with pipetting, the bone marrow cells were centrifuged and counted using a LUNA-II automated cell counter. For hematopoietic cell analyses, cell suspensions from spleens (crushed between two frosted ends of tissue slides and filtered) and bone marrow were first incubated with anti-mouse Ly76 mAb and, after washing, were detected by FITC-conjugated goat anti-rat IgG as secondary reagent in PBS containing 0.1% BSA and Na-azide on ice for 20 min. After washing, the cells were incubated in 50-fold diluted normal rat serum to saturate remaining binding sites of secondary reagent for rat IgG, followed by the addition of rat anti-mouse CD45 mAb labeled with Alexa Fluor 647 dye. For LEC analysis, lymph nodes or spleens from Prox1GFP-Nkx2-3−/− mice were digested with a cocktail of DNAse I/Liberase (Roche) for 20 min at 37°C in DMEM. Released cells were incubated with a cocktail of PE anti-mouse gp38, PerCP-Cy5.5 anti-mouse CD45, and APC anti-mouse CD31, supplemented with 7-AAD to exclude dead cells. After washing, 10,000 events gated on forward light scatter (FSC)/side light scatter (SSC) parameters (or for LECs, excluding CD45-positive/7-AAD-positive cells) were collected and analyzed using BD FACSCalibur and CellQuest Pro software package or FlowJo v10.

### 2.4 Immunofluorescence and immunohistochemistry

After snap-freezing of spleen tissues embedded in Killik medium, 8 µm cryosections were prepared and acetone fixed. For immunofluorescence the sections were saturated with 5% BSA, followed by incubation with IBL-9/2, anti-Clever1 or anti-VCAM-1 mAbs against murine endothelial cells or TER-119 mAb against Ly76 for 45 min, revealed by subsequent incubation with FITC-conjugated goat anti-rat IgG or Cy3-conjugated goat anti-rat IgG. Next, 50-fold diluted normal rat serum was applied to saturate remaining anti-rat IgG, followed by incubation with IBL-9/2 conjugated with Alexa Fluor-647 and FITC-conjugated anti-CR1/2, or biotinylated anti-CD29 mAb and anti-CD45 mAb conjugated with Alexa Fluor 647, respectively. Finally, the binding of biotinylated anti-CD29 mAb was revealed by incubating the sections with PE-streptavidin. For immunohistochemistry, fixed and dried sections were treated with 0.1% phenyl-hydrazine to block endogenous peroxidase, followed by washing and saturation with 5% BSA. The sections were then incubated with anti-Clever1 or IBL-9/2 or anti-CD41 mAbs, which were visualized by subsequent incubation with ImmPress goat anti-rat IgG peroxidase conjugate and color development using DAB-H_2_O_2_ substrate mixture and counterstained with Mayer’s hematoxylin. After mounting, the sections were viewed under an Olympus BX61 microscope, digital images were taken using the ZEN software and processed using Photoshop. For quantification sections 200 µm apart were evaluated by Pannoramic View scanner (3D Histech, Budapest, Hungary) and QuPath-0.2.3 software. Whole-mount spleen samples from Prox1GFP-Nkx2-3−/− or heterozygote control mice were fixed in 4% buffered paraformaldehyde, followed by CUBIC optical clearing procedure (Susaki et al., 2014). Whole-mount samples were visualized by confocal microscopy using an Olympus FluoView FV1000 laser scanning confocal imaging system (Olympus Europa SE & Co., Hamburg, Germany). Images were acquired using a ×20 dry objective (NA: 0.75), GFP was excited by a 488 nm laser line and fluorescence emission was detected between 500 and 600 nm. The 3D images were rendered in the Imaris Software (Bitplane AG, Zurich, Switzerland) using Z-stack data.

### 2.5 Blood count parameters and serum cytokine detection

Approximately 150–200 µL blood was collected in K3 EDTA 0.25/0.5 ml MiniCollect Complete tubes. The blood parameters were measured in a Sysmex XN-V1000 automated hematology analyzer and its software package. Serum erythropoietin was measured using the Legend Max mouse EPO ELISA kit (BioLegend, from Biomedica Hungaria, Budapest, Hungary).

### 2.6 Statistical analysis

The data were analyzed using GraphPad Prism 9 software. Significance between groups was determined by performing either a Mann-Whitney *U* test or a paired *t*-test, with significance levels defined as *p < 0.05, **p < 0.01, ***p < 0.001, and ****p < 0.0001.

## 3 Results

### 3.1 Appearance of ectopic *bona fide* lymphatic vessels in Nkx2-3^−/−^ spleen

**FIGURE 1:**
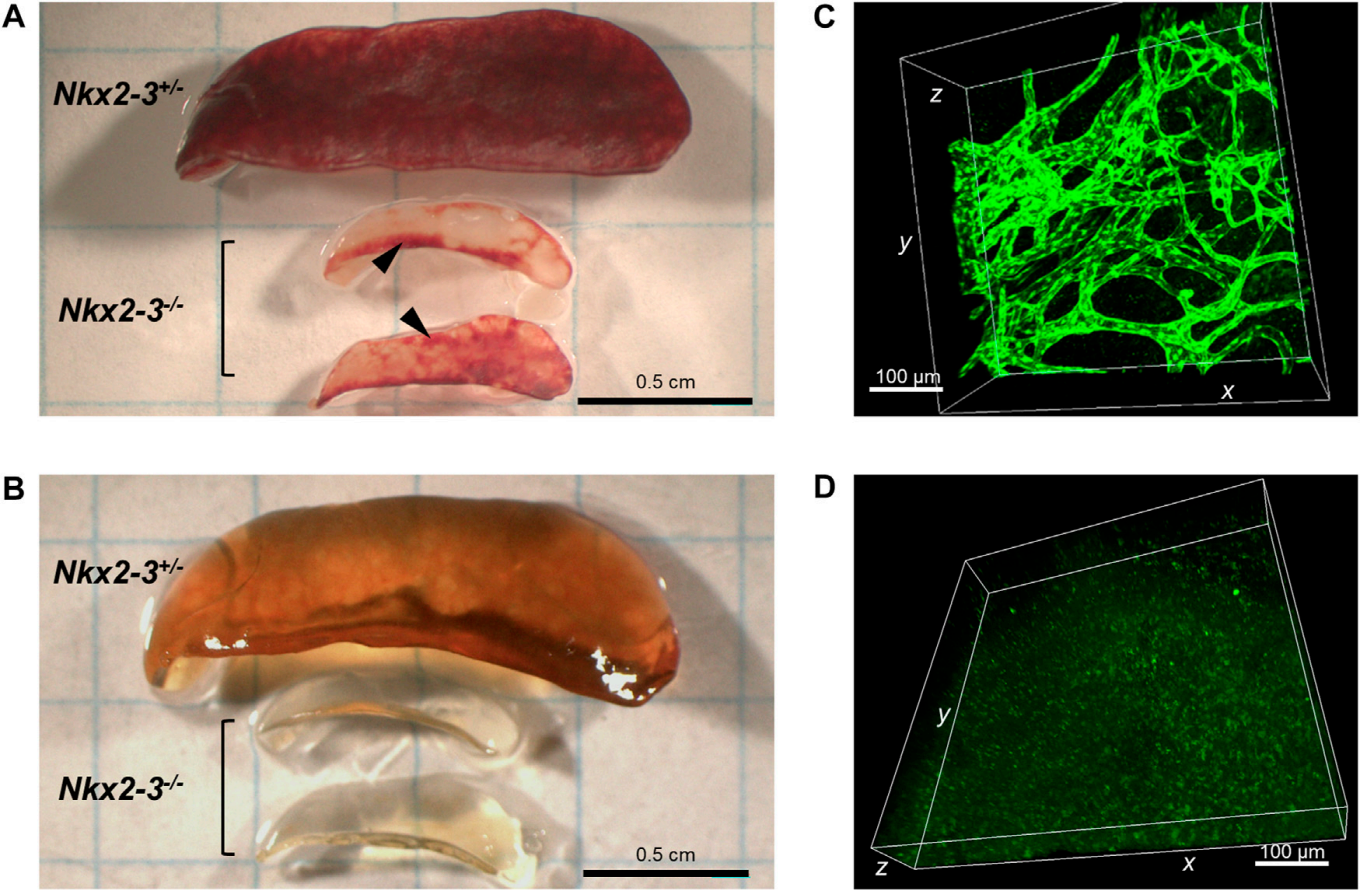
Organziation of Prox1-expressing lymphatic capillaries in Nkx2-3^−/−^ spleens. Compared to heterozygote sample (top) Nkx2-3-deficient mice have a reduced spleen size and patchy red pulp appearance (middle and bottom) **(A)**. Following paraformaldehyde fixation and transparency treatment of the same samples, the heterozygote tissue (top) could not be clarified due to residual hemoglobin pigment, while the homozygote KO samples (middle and bottom) have become completely transparent **(B)**. After CUBIC treatment in *Prox1* ^*GFP*^-Nkx2-3^−/−^ mice **(C)** an extensive meshwork of LEC capillaries can be observed, while in *Prox1* ^*GFP*^-Nkx2-3^+/-^ **(D)** such structures are absent, only faint background autofluorescence is detectable. Scale bars in A-B = 0.5 cm; scale bars in C-D = 100 μm. Representative images of a cohort of n = 3, experiment repeated twice.

**FIGURE 2:**
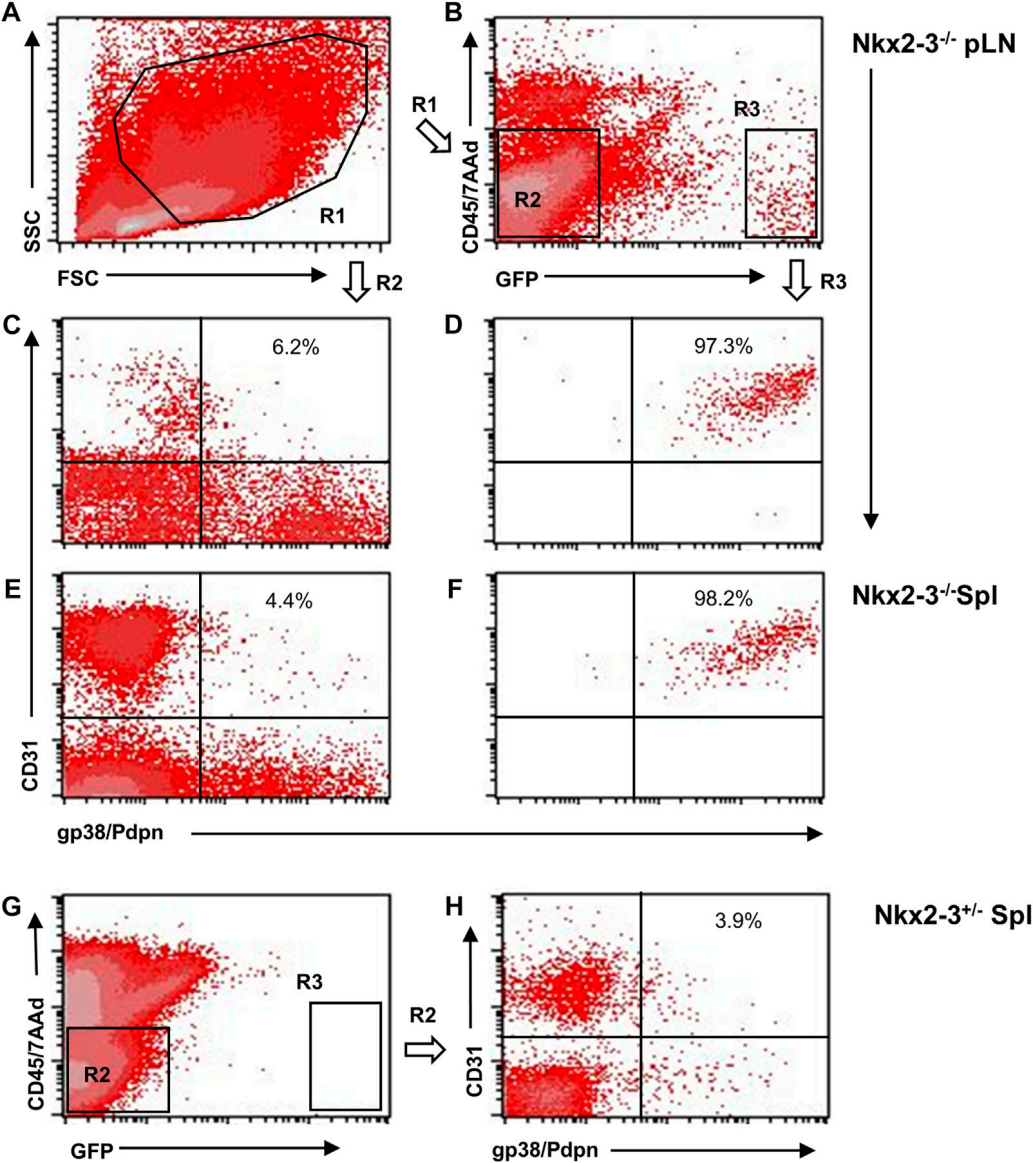
Flow cytometric characterization of Prox1:eGFP^+^ cells in mice lacking Nkx2-3. Peripheral lymph nodes (pLN) were gated to include large stromal cells [**(A)**, R1] and either eGFP^−^ [**(B)**, R2/left] or eGFP^+^ [**(B)**, R3/right] cells, then resolved according to their gp38(podoplanin)/CD31 features. Representative images demonstrate that the Prox1:eGFP^+^ (R3) cells in both pLN **(D)** and spleen (Spl, **(F)** from Nkx2-3^−/−^ mice display gp38/CD31 double positive LEC phenotype (numbers indicating their frequency), whereas eGFP:Prox1^-^ cells from Nkx2-3^−/−^ pLN **(C)** and spleen **(E)** constitute non-LEC stromal subsets. In Nkx2-3^+/-^ spleens no GFP^+^ signal is present **(G)**. **(H)**, gp38(podoplanin)/CD31 staining of GFP^−^ (R2) cells in Nkx2-3^+/-^ spleens. Representative dot plots from n = 4, experiment repeated twice.

Previously we found that, in addition to the lymph node-like shift of addressin preference from MAdCAM-1 in marginal sinus-lining cells to PNAd displayed by ectopic high endothelial venules (HEVs) (Czömpöly et al., 2011), the spleens of Nkx2-3-deficient mice also contain lymphocyte-filled vascular sacs expressing LYVE-1 LEC-associated hyaluronan receptor, surrounded by a fibroblastic meshwork identifiable by ER-TR7 mAb against Collagen type VI (Kellermayer et al., 2011; Schiavinato et al., 2021). As LYVE-1 can also be expressed by non-LECs, such as serosal macrophages or splenic megakaryocytes (Schledzewski et al., 2006), and our previous anti-Prox1 immunohistochemical results provided conflicting results, next we also tested Prox1 expression in Nkx2-3-mutant spleens as master regulator for LEC specification by backcrossing Nkx2-3^−/−^ mice with Prox1GFP mice. First, we investigated whole-mount spleen samples from Nkx2-3 deficient and heterozygote mutant mice, using the CUBIC optical clearing procedure (Susaki et al., 2014). We found that this treatment effectively rendered the Prox1GFP-Nkx2-3^−/−^ spleens completely transparent, while in heterozygote and wild-type (not shown) samples some residual brownish hemoglobin-derived pigment remained (Figures 1A,B). In Prox1GFP-Nkx2-3^−/−^ mice, we found an extensive arborized meshwork of Prox1-positive tubes (Figure 1C and Supplementary Movie S1), with their diameter ranging between 15 and 40 μm. These structures often created anastomoses between branchings, establishing ring-like connections, but revealed no valve-like formations, indicating initial lymphatic capillaries (Lutter et al., 2012). In contrast, in Nkx2-3 heterozygote spleens with Prox1 reporter expression we only occasionally observed low-level GFP^+^ signal associated either to macrophages or due to background autofluorescence, without any evidence for capillary formation (Figure 1D). Next, we used flow cytometry to confirm that, in Prox1GFP-Nkx2-3^−/−^ mice, the splenic GFP^+^ cells are identifiable as LECs (Figure 2). Indeed, GFP^+^ cells were CD45^-^gp38^+^CD31^+^, corresponding to LECs normally present in lymph nodes (Link et al., 2007) (Figure 2D). In contrast, the GFP^−^CD45^−^ cells (also excluding the 7AAD-positive dead cells) included gp38^−^CD31^−^, and gp38 or CD31 single positive mesenchymal cells, corresponding to the follicular or other stromal cells including follicular dendritic cell (FDCs) and pericytes, T-zone reticular fibroblasts (FRCs) or blood endothelial cells (BECs) as main stromal subsets, respectively (Figures 2C,E,G). With these flow cytometric settings, we were unable to identify any GFP^+^CD45^−^ live cells in spleens of Nkx2-3 heterozygous mice (Figure 2H). These finding indicate that in *Prox1*-GFP^+^ Nkx2-3-deficient spleens, gp38^+^/CD31^+^ double positive LECs are the only GFP-positive cells. Based on these observations, we conclude that the RP vasculature of the spleen in the absence of Nkx2-3 is transformed to harbor ectopic lymphatic capillary meshwork with gp38/CD31 double positive cell surface phenotype and Prox1-expression corresponding to LECs.

### 3.2 Absence of Nkx2-3 causes defective red pulp vascular specification of the spleen

**FIGURE 3:**
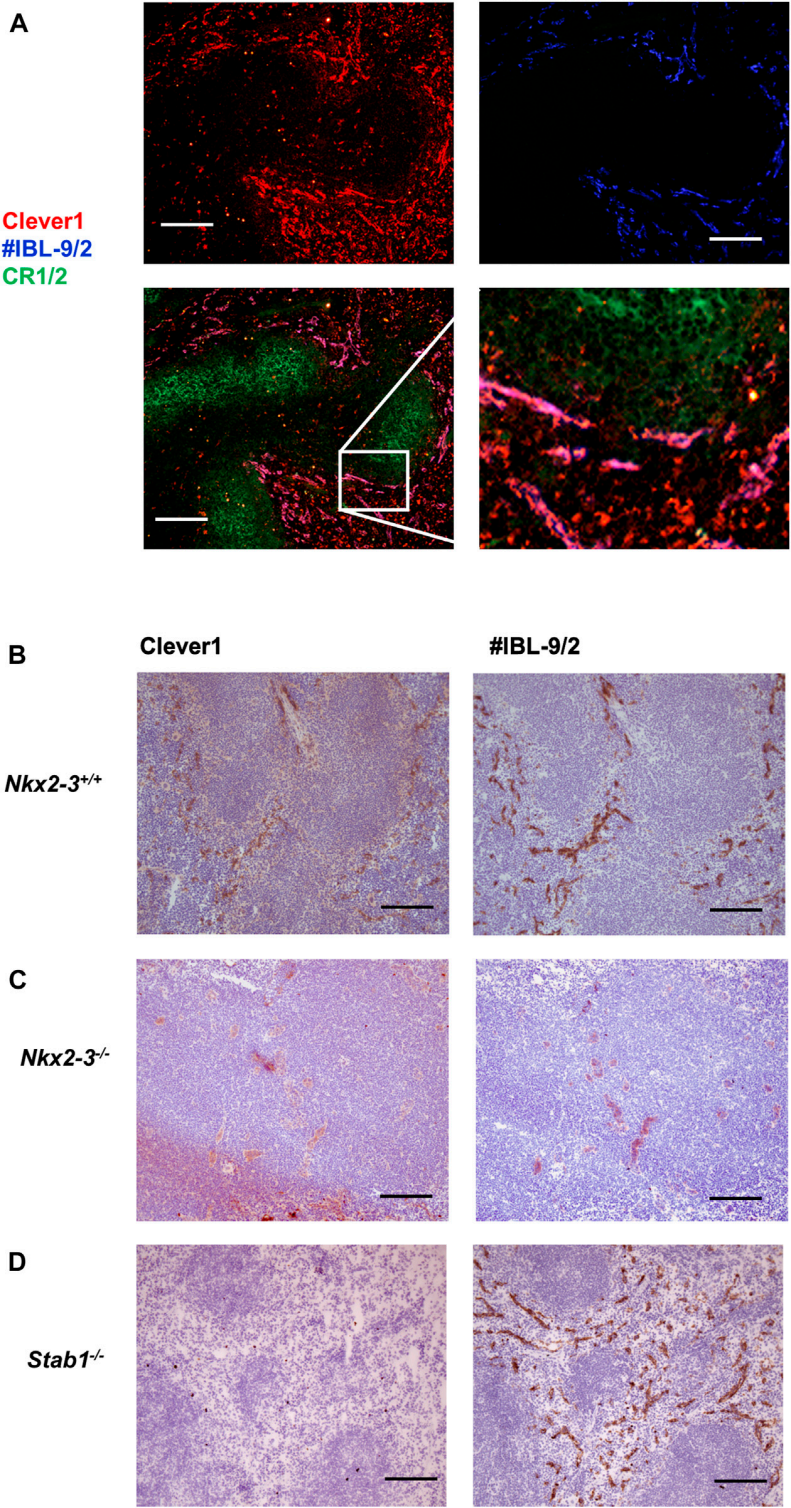
Nkx2-3 is necessary for the spleen red pulp vascular specification. Cryosections from C57Bl/6J mice were stained with the indicated antibodies, demonstrating co-localization of Clever1 (red) and IBL-9/2 (blue) staining (top) with relation to white pulp follicular dendritic meshwork (CR1/2, green) to visualize follicles (bottom, left) in wild-type spleen. Inset of the merged image demonstrates the vascular overlap between Clever1 and IBL-9/2 markers as purple **(A)**. In wild-type mice **(B)** both Clever1 and IBL-9/2 labeling are present, whereas the absence of Nkx2-3 leads to the absence of both markers **(C)**; in contrast, the deletion of Stab1 only abolishes Clever1 expression **(D)**. Representative images, experiment repeated twice, *n* = 4, scale bar = 100 µm.

The presence of lymphatic capillaries in the spleen of mice lacking Nkx2-3 suggests a major shift in the splenic vasculature, typically lacking such structures in wild-type mice. We have previously observed a significant reduction of red pulp (RP) venous sinus vessels identifiable with IBL-9/2 mAb (Balogh et al., 2007). To further examine how vascular alterations in this genotype affect other specific traits of this regional vasculature, next we investigated the expression of Clever1 scavenger receptor encoded by Stab1 gene. This endocytic receptor on LECs in wild-type mice is also expressed by non-continuous BECs involved in splenic B-cell homing to RP (Prevo et al., 2004; Tadayon et al., 2019). Using immunofluorescence microscopy, we found that in wild-type mice, Clever1 expression in the RP vasculature largely overlaps with the reactivity of IBL-9/2 (Figures 3A,B), whereas the absence of Nkx2-3 abolished the sinusoidal labeling for both markers (Figure 3C). In contrast, although Stab1^−/−^ mice lack Clever1, the RP sinus reactivity of IBL-9/2 mAb remained unaltered (Figure 3D). These findings indicate that the vascular shift elicited by the absence of Nkx2-3 affects both the maturation of RP sinus meshwork containing BECs, and the differentiation of ectopic LEC capillaries into Clever1^+^ segments.

### 3.3 Disrupted vascular stroma in Nkx2-3-deficient spleen impairs extramedullary stress hematopoiesis

**FIGURE 4:**
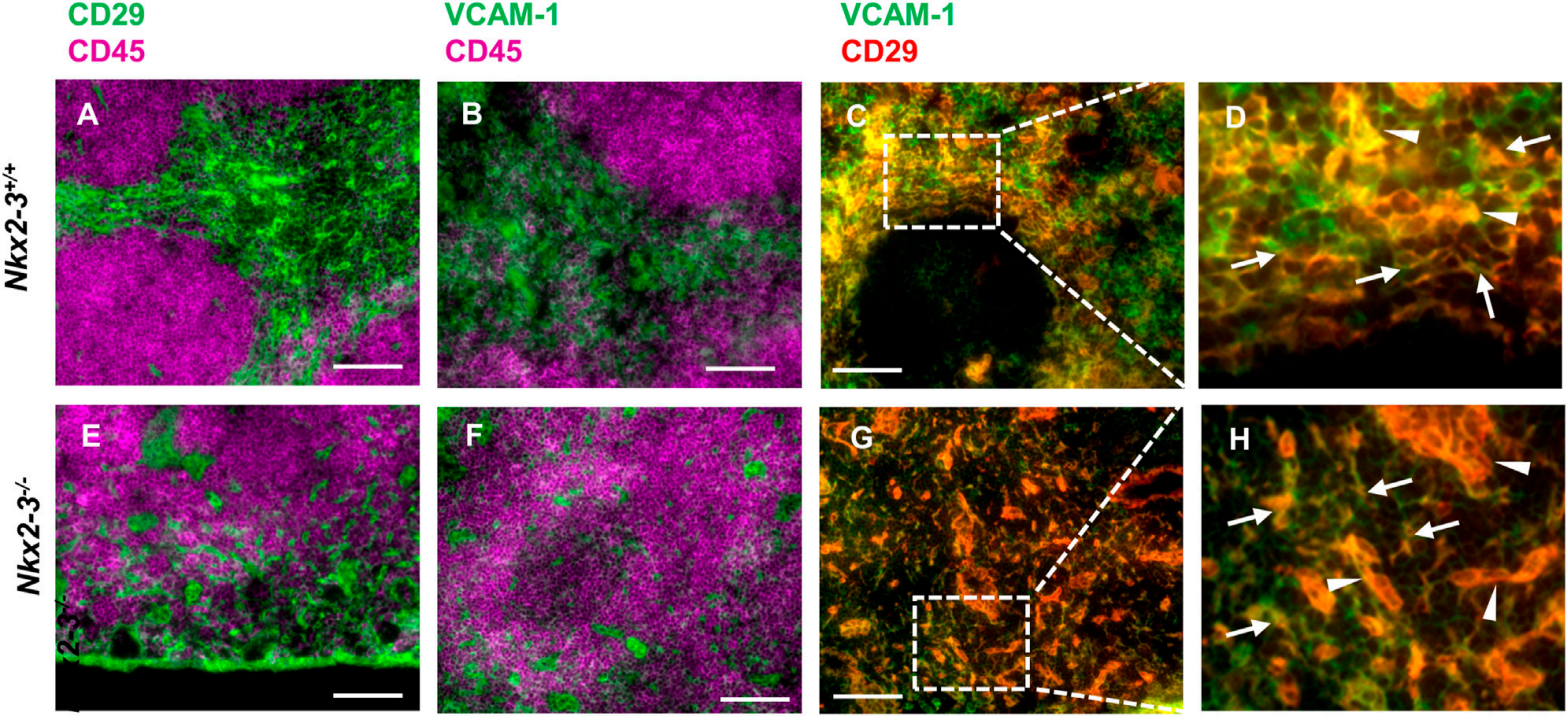
Hematopoiesis-supporting splenic stroma is disrupted in Nkx2-3^−/−^ mice. Cryosections from Nkx2-3^+/+^ (**A–D**) and Nkx2-3^−/−^ spleens (**E–F**) were stained for indicated markers, revealing intense expression of both CD29 and VCAM-1 (green) outside the white pulp delineated by CD45 staining (purple). Higher power images of the rectangles within the RP marked [in **(C, G)**] with dashed line demonstrate both stromal (arrows) and vascular (arrowheads) co-expression of CD29 and VCAM-1 [appearing as yellow, **(D, H)**]. Representative dual fluorescence microscopic images from a cohort of *n* = 4. Scale bars correspond to 100 μm.

In mice the RP vasculature plays an important role in extramedullary hematopoiesis as RP sinusoidal endothelial cells, in association with Tcf21^+^ stromal cells, create a niche for hematopoietic stem cells by providing them with stem cell factor/KitL and CXCL12 (Inra et al., 2015). As in Nkx2-3^−/−^ spleens we observed a defective RP vasculature and ectopic lymphatics, we were interested in whether this shift has any effect on the hematopoiesis-supporting stroma. Although the various stromal components in the splenic RP are not as clearly delineated phenotypically as in the white pulp (Lim and O'Neill, 2019; Bellomo et al., 2020), the extramedullary hematopoietic activity of the splenic RP has been suggested to involve CD29-positive stromal cells (O'Neill et al., 2019). Moreover, the RP reticular stroma as well as endothelial cells also broadly display VCAM-1. Therefore, next we investigated the presence of stromal cells by multicolor immunofluorescence of these stroma markers. We found that both CD29 and VCAM-1 in normal BALB/c mice were expressed dominantly by RP stromal constituents (Figures 4A,B), in addition to blood vessels. The CD29^+^/VCAM-1^+^ extravascular stromal cells form a meshwork throughout the RP (Lim and O'Neill, 2019; O'Neill et al., 2019) (Figures 4C,D). In Nkx2-3 mutants, we could also detect CD45-positive regions intermingled with both vascular and non-vascular CD29^+^/VCAM-1^+^ cells (Figures 4E,F). However, in Nkx2-3-deficient mice, we observed that although both stromal and vascular CD29^+^/VCAM-1^+^ cells are present (Figures 4G,H), the RP stromal compartment is fragmented, without a definable red pulp-white pulp boundary.

Next, we investigated whether the vascular-stromal alterations observed in the RP affect the reserve hematopoietic potential of the spleen. First, we measured basic blood parameters to obtain a general overview of the hematopoietic function of mice lacking Nkx2-3. These parameters revealed chronic anemia in Nkx2-3 deficient mice, manifested as significantly lower red blood cell (RBC, *p = 0.008*), hematocrit (HCT, *p = 0.0143*) and hemoglobin (HGB, *p = 0.0073*) levels in Nkx2-3^−/−^ mice compared to wild type BALB/c mice (Supplementary Figures S1A–C). In contrast, the absolute number of total white blood cells, including both neutrophils (*p = 0.0007*) and lymphocytes (*p = 0.008*) was significantly increased (Supplementary Figures S1D–F). Blood loss induced by repeated blood withdrawals led to significantly lower RBC (*p = 0.02*) and HGB (*p = 0.0135*) levels in BALB/c mice, while the hematocrit was unchanged. In Nkx2-3^−/−^ mice, we observed a similar pattern of moderately reduced hematocrit without reaching statistically significant difference, but with significantly lower RBC (*p = 0.0303*) and HGB (*p = 0.0173*) levels. Importantly, in treated Nkx2-3^−/−^ mice all three values were significantly lower, compared to treated wild-type mice (Supplementary Figures S1A–C). To rule out that in Nkx2-3-deficient mice decreased EPO production is responsible for reduced erythropoiesis, we compared EPO levels in untreated and bled BALB/c and Nkx2-3 mutant mice by ELISA. We found that in untreated mice lacking Nkx2-3 the basal EPO level was significantly higher compared to BALB/c mice (*p = 0.0011*), and upon blood loss, this further increased, significantly exceeding that of treated BALB/c mice (Supplementary Figure S1G). This finding also indicates that under steady-state conditions, the increased level of EPO is insufficient to maintain a normal hematocrit level, and the significantly augmented EPO production fails to achieve erythropoietic recovery in mice lacking Nkx2-3.

**FIGURE 5:**
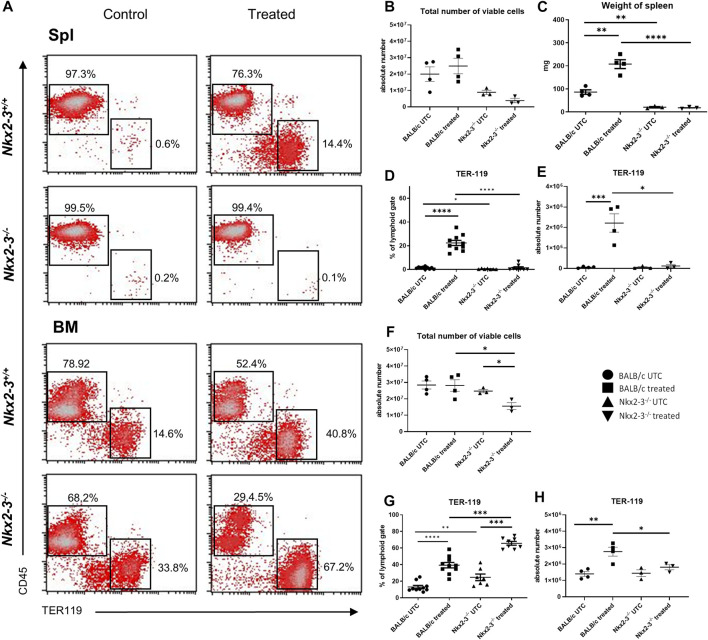
Acute blood loss in Nkx2-3^−/−^ mice induces imbalanced erythropoietic responses in the bonee marrow and spleen. Representative flow cytometric plots demonstrate results of the distribution of bone marrow and spleen lymphoid and erythroid cells in BALB/c and Nkx2-3^−/−^ UTC and treated mice, numbers indicate relative frequency in the rectangles **(A)**. Total number of lymphoid gated spleen cells in BALB/c (UTC n = 4, treated n = 4) and Nkx2-3^−/−^ (UTC n = 3, treated n = 3) mice **(B)**. Wet weight of spleen in BALB/c (UTC n = 4, treated n = 4) and Nkx2-3^−/−^ (UTC n = 3, treated n = 3) mice **(C)**. Ratio of splenic erythroid cells in BALB/c (UTC n = 10, treated n = 10) and Nkx2-3^−/−^ (UTC n = 7, treated n = 8) mice **(D)**. Absolute number of splenic erythroid cells in BALB/c (UTC n = 4, treated n = 4) and Nkx2-3^−/−^ (UTC n = 3, treated n = 3) mice **(E)**. Total number of lymphoid gated viable bone marrow cells in BALB/c (UTC n = 4, treated n = 4) and Nkx2-3^−/−^ (UTC n = 3, treated n = 3) mice **(F)**. Ratio of bone marrow erythroid cells in BALB/c (UTC n = 10, treated n = 10) and Nkx2-3^−/−^ (UTC n = 7, treated n = 8) mice **(G)**. Absolute number of bone marrow erythroid cells in BALB/c (UTC n = 4, treated n = 4) and Nkx2-3^−/−^ (UTC n = 3, treated n = 3) mice **(H)**. *p < 0.05, **p < 0.01, ***p < 0.001, ****p < 0.0001 by unpaired *t*-test and Mann Whitney *U* test.

To define the extramedullary erythropoietic capacity in spleen, the frequency and absolute number of splenic TER-119^+^/CD45^-^ erythroid lineage cells were determined by flow cytometry in Nkx2-3^−/−^ and wild-type BALB/c mice, and compared to bone marrow after repeated bleeding (Figure 5A). We found that in untreated Nkx2-3^−/−^ mice the frequency and absolute number of splenic erythroid cells were lower compared to BALB/c mice. After bleeding the spleen in Nkx2-3 mutant mice failed to reactivate its erythropoietic activity, while in BALB/c mice a robust erythroid expansion occurred (Figures 5B–E). In the bone marrow, the total cell number was lower in Nkx2-3^−/−^ mice compared to BALB/c mice, and following bleeding it did not change significantly in BALB/c mice, whereas in Nkx2-3^−/−^ mice the total cell number significantly (*p = 0.0189*) decreased (Figure 5F). In bone marrow the ratio of TER-119-positive erythroid cells was significantly higher in untreated Nkx2-3^−/−^ mice than in wild-type controls (*p = 0.0079*), and their frequency significantly increased in both genotypes following bleeding (Figure 5G). In Nkx2-3-deficient mice this increase of TER-119-positive cell percentage was significantly higher; however, the increase in absolute numbers was significant (*p = 0.0046*) only in BALB/c mice (Figure 5H).

**FIGURE 6:**
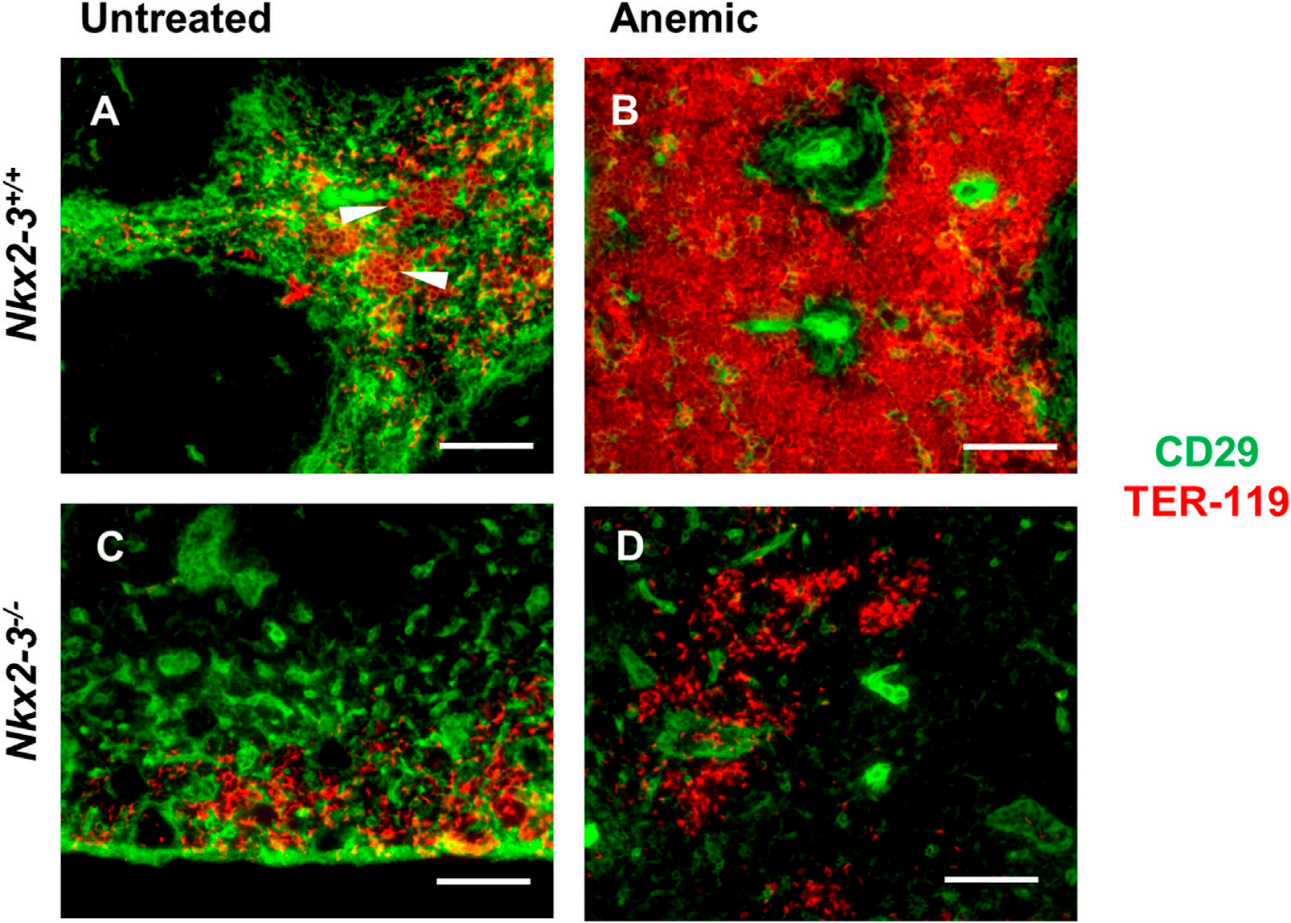
Defective extramedullary expansion of splenic erythroid colonies in mice lacking Nkx2-3 compared to wild-type mice. Compared to untreated controls **(A)**, in anemic BALB/c mice the TER-119-positive erythroid colonies (red, arrowheads) embedded in CD29-positive stroma (green) expand throughout the RP following blood withdrawal **(B)**; in Nkx2-3^−/−^ mice **(C–D)** only erythrocyte clusters are visible, without any noticeable change upon blood loss. Representative dual fluorescence microscopic images from a cohort of n = 4. Scale bars correspond to 100 μm.

Immunohistochemical analyses in untreated BALB/c mice revealed TER-119-positive erythroid clusters within the red pulp-restricted CD29-positive stromal meshwork, in addition to the erythrocytes trapped in the RP (Figure 6A); however, such clusters were absent in Nkx2-3-deficient spleen, only erythrocyte clumps were present (Figure 6C). Following blood withdrawal, in BALB/c mice the TER-119-positive erythroid colonies become confluent, virtually completely filling the tissue space between the neighboring white pulp regions (Figure 6B). In contrast, in Nkx2-3-deficient mice following the loss of blood the expansion of RP TER-119-positive colonies was completely abolished, although CD29-positive extravascular reticular cells were present, indicating the tissue-specific functional impairment of erythropoietic support (Figure 6D). These results establish that, in addition to the reduced steady-state splenic erythropoiesis, the induced stress hematopoiesis is also significantly impaired in Nkx2-3 deficient mice in a tissue-specific manner.

### 3.4 Absence of Nkx2-3 blocks the splenic megakaryocyte expansion following TPO-agonist treatment

In addition to the capacity of producing erythroid cells, the splenic RP in mice also harbors megakaryocytes, as a RP-dwelling non-lymphoid resident hematopoietic lineage (Noetzli et al., 2019). To test if the defective extramedullary hematopoiesis also affects the occurrence of splenic megakaryocytes and their expansion, we next investigated the effect of intravenously injected Romiplostim, a peptide-fusion protein acting as TPO receptor agonist stimulating megakaryocytes (Léon et al., 2012). The effect was determined by the morphometric quantification of the splenic megakaryocyte numbers, following immunohistochemical staining for CD41.

**FIGURE 7:**
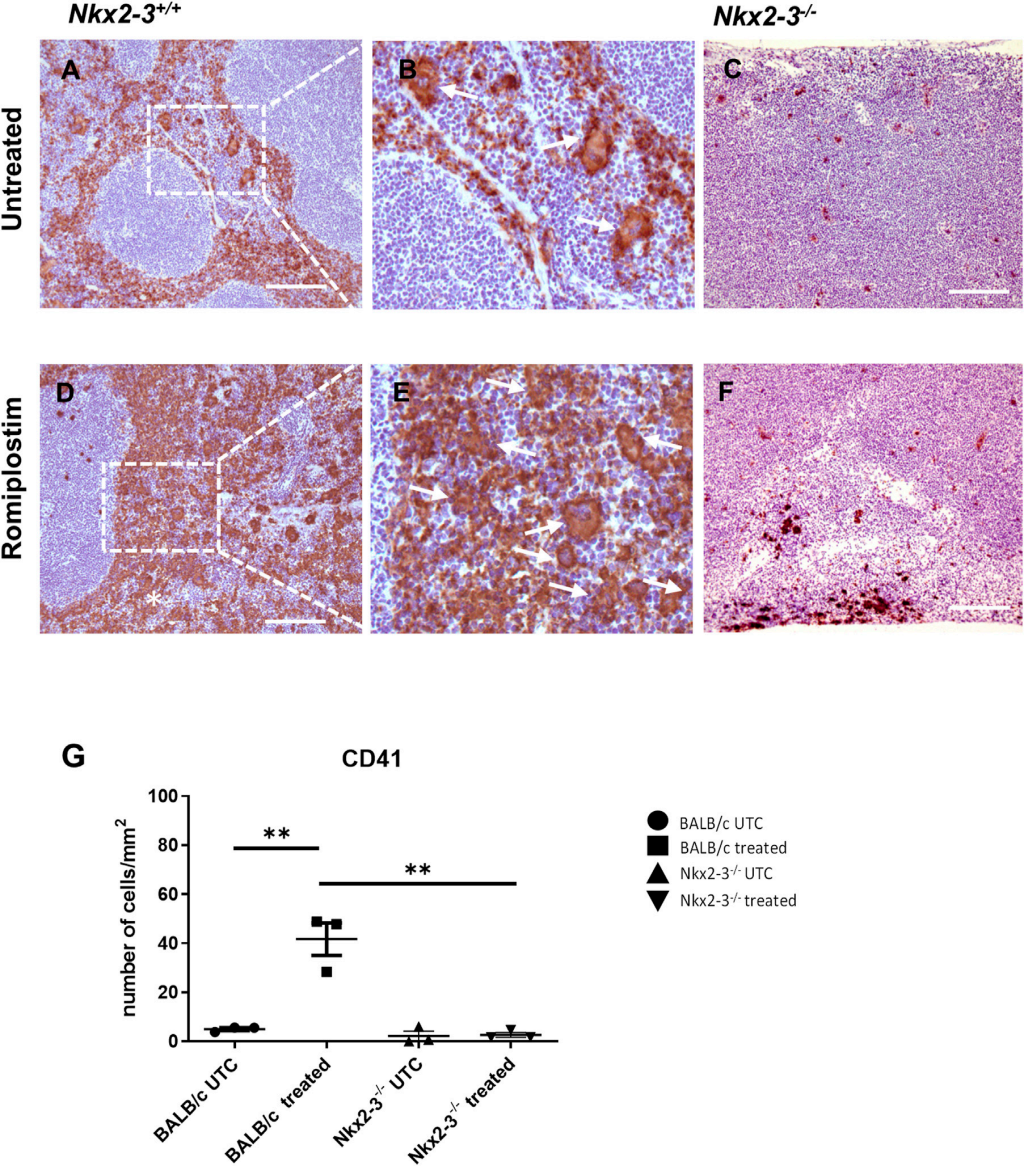
TPO-agonist treatment fails to induce splenic megakaryocyte expansion in mice lacking Nkx2-3. Representative images show megakaryocyte labeling of BALB/c or Nkx2-3^−/−^ (indicated at the top) spleen using anti-CD41 immunohistochemistry (visualized as brown reaction product, with hematoxylin counterstain) before (untreated, **(A–C)** and after Romiplostim treatment **(D–F)**, indicated on the left. Higher power inserts **(B, E)** corresponding to the dashed rectangle demonstrate single megakaryocytes restricted to the RP. In the samples after Romiplostim treatment the arrows in the high-power insert (corresponding to the rectangle) label multinucleated megakaryocytes typically arranged in pairs. Representative micrographs from a cohort of *n* = 4. Scale bars correspond to 100 μm. Graphs demonstrate the number of CD41^+^ cells per 1 mm^2^ in BALB/c (UTC n = 3, treated n = 3) and Nkx2-3^−/−^ (UTC n = 3, treated n = 3) mice **(G)**. **p < 0.01 by unpaired *t*-test and Mann Whitney *U* test **(G)**.

In untreated BALB/c mice, megakaryocytes in the RP localized frequently in the subcapsular regions as single cells or doublets (Figures 7A,B). In contrast, in Nkx2-3-deficient mice only occasional megakaryocytes were present, also typically in subcapsular locations (Figure 7C). Romiplostim treatment led to a robust increase of platelet-associated labeling by anti-CD41 staining in wild-type spleens (Figures 7D,E) significantly blurring the identification of megakaryocytes. We observed only minimal increase in Nkx2-3-deficient spleens (Figure 7F). Quantification of megakaryocytes by Pannoramic Viewer scanner and QuPath-0.2.3 software based on their nuclear morphology revealed an approximately tenfold increase for megakaryocyte number in BALB/c spleen (*p = 0.0053*) (Figure 7G). In contrast, such an increase was absent from Nkx2-3-deficient mice. Together these data indicate that while the residual RP in Nkx2-3-deficient mice may harbor a reduced number of megakaryocytes, the spleen cannot expand its megakaryocyte population following TPO-receptor agonist stimulation, in contrast to wild-type BALB/c mice.

## 4 Discussion

In this work, we describe the identification of ectopic LEC capillaries displaying lymphatic endothelial cell (LEC) surface markers and Prox1 fate-determining transcription factor in splenic vasculature in mice lacking Nkx2-3, coupled with defective stromal differentiation and support capacity of splenic stress hematopoiesis. These observations confirm Nkx2-3 as a critical factor for both tissue-specific lymphatic-blood endothelial commitment as structural elements, and as essential components to maintain the stress hematopoietic capacity of spleen.

After the separation of splanchnic mesenchyme, the emerging spleen anlage is arranged around its blood vasculature, surrounded by specialized stromal constituents, also accompanied by regional macrophage subsets (Steiniger et al., 2007; Bellomo et al., 2021). Recent analyses of splenic stromal subpopulations using scRNAseq combined with lineage tracing and phenotypic identification have revealed substantial functional differences coupled with complex tissue location preferences, particularly for white pulp fibroblastic reticular cell subsets (Cheng et al., 2019; Alexandre et al., 2022). Typically, these analyses address the reticular scaffolding of the various splenic compartments, although the blood endothelial subsets also display considerable heterogeneity, including phenotypic markers, chemokine profile and adhesion molecules (Balázs et al., 2001; Berahovich et al., 2014; Tadayon et al., 2019). Despite the earlier observations demonstrating the presence of deep efferent lymphatic vessels in mice (Pellas and Weiss, 1990; Steiniger and Barth, 2000), analyses addressing the origin of putative splenic LECs are scarce. The exact origin of these splenic lymphatic vessels in humans is currently unknown, most likely owing to their much less obvious presence (through the use of lymphatic markers including LYVE-1, podoplanin or Prox1) compared to other lymphoid tissues, such as lymph nodes, Peyer’s patches, or dermal and intestinal lymphatics.

In agreement with previous phenotypic and structural analyses on the tissue-specific role of Nkx2-3 in defining the spleen’s vasculature, comparison of splenic stromal subsets and the corresponding lymph node-derived subsets using scRNAseq approach also demonstrated that Nkx2-3 is amongst the most distinctive genes characteristic for the spleen (Alexandre et al., 2022). Its mRNA expression is also shared by various splenic white pulp reticular cell subpopulations—comprised of at least eight different mesenchymal subsets, in addition to vascular-related adventitial and pericyte as well as mesothelial cells - located within the white pulp and marginal zone. In contrast, the red pulp stromal subsets (while constituting a large part of total splenic stromal cells) were less divergent, and analyzing a public database (accessible at http://muellerlab.mdhs.unimelb.edu.au/frc_scrnaseq/) for their Nkx2-3 expression presents only a moderate expression of Nkx2-3.

Earlier immunohistochemical findings from our laboratory demonstrate that in human samples the expression of Nkx2-3 is restricted to RP endothelial cells (Kellermayer et al., 2016). This distribution mirrors the drastic reduction of RP vessels identifiable by IBL-9/2 mAb and displaying Clever1 (encoded by *Stab1*) in Nkx2-3 deficient mice. Recent comprehensive expression analyses reveal that Nkx2-3 mRNA is selectively expressed in splenic, small intestinal and colonic vascular endothelium (Kalucka et al., 2020). Interestingly, however, neither *Nkx2-3* nor *Stab1* is included in the top-50 expressed genes in various vascular segments of the spleen, while *Prox1* is listed, but without *Lyve1*. Our present findings demonstrate evidences that RP blood vessels are largely replaced by ectopic Prox1-positive lymphatic capillaries, indicating a defining role for Nkx2-3 in vascular patterning (including lymphatic vs. blood endothelium commitment) of the spleen.

**FIGURE 8:**
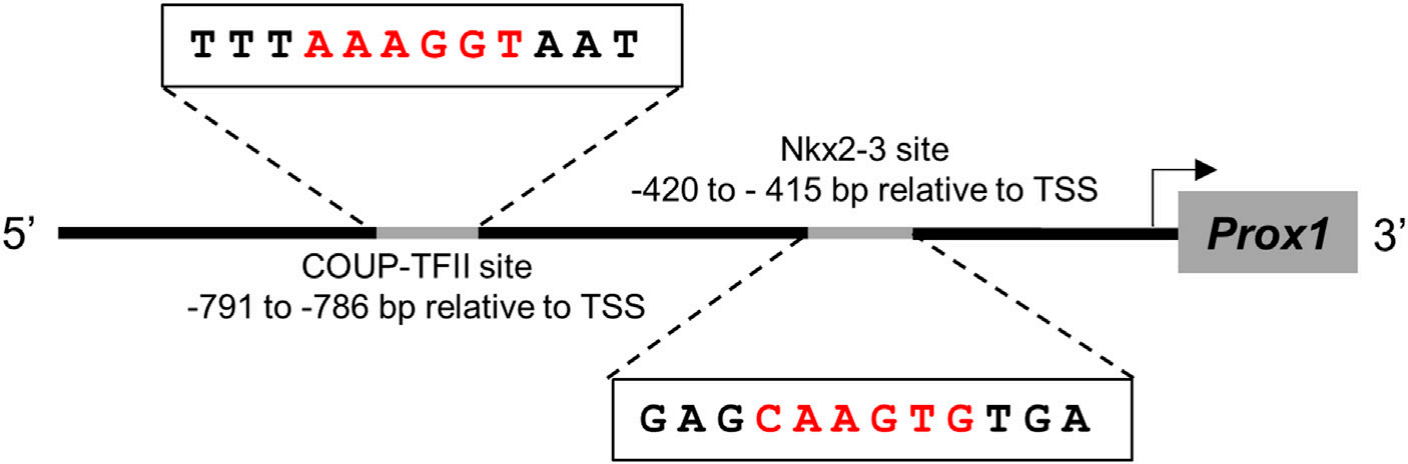
Sequence analysis of Prox1 promoter for putative binding sites for Nkx2-3 and COUP-TFII The transcription factors are indicated at the top, their binding sequence motifs are demonstrated according to JASPAR database, compared to the putative target sequences within the promoter and their sequence relative to transcription start site (TSS).

What can the explanation be for this eventual shift of RP vasculature into putative lymphatic capillaries? In the HEVs of intestinal lymphoid tissue vasculature, Nkx2-3 activates the promoter of MAdCAM-1 addressin through forming a composite element together with DNA-binding orphan nuclear receptor COUP-TFII, a critical transcription factor defining vein identity through suppressing Notch activity (You et al., 2005; Dinh et al., 2022). This mechanism relies on the availability of respective binding sites for both Nkx2-3 and COUP-TFII within the MAdCAM-1 promoter region in HEV-programmed venous endothelial cells. As Prox1 also exerts its LEC-defining activity in venous endothelial cells (Wigle and Oliver, 1999), it is tempting to speculate that a similar dual regulation may take place in the spleen, where Nkx2-3 may inhibit the expression of Prox1, while COUP-TFII continues to define venous specification. Indeed, search of promoter database (https://epd.epfl.ch) pinpoints potential bindings sites for both Nkx2-3 and COUP-TFII in the Prox1 promoter region (Figure 8). It remains to be investigated whether Nkx2-3, together with COUP-TFII (thus in a vein-specific manner) may influence Prox1 expression, thereby pivoting the sinus vein endothelium towards LEC fate commitment, in addition to the altered postnatal blood endothelial addressin preference from MAdCAM-1 to PNAd upon lymphocyte accumulation (Mebius et al., 1996; Balázs et al., 2001; Balogh et al., 2007; Zindl et al., 2009).

Concerning the functional consequences of this shift, we have observed a profound defect of extramedullary hematopoiesis along both the erythroid and megakaryocyte lineages, even though phenotypic analyses indicate the presence of non-endothelial CD29^+^Sca-1^+^gp38^+^Thy1.2^+^CD51^+^ positive splenic stromal cells which are also present in wild-type mice and are instrumental for RP-associated hematopoiesis (Lim and O'Neill, 2019). As the bone marrow erythropoiesis could be augmented following blood loss in both wild-type and Nkx2-3 mutants, the lack of Nkx2-3 is unlikely to cause the failure of hematopoietic stem cells to promote extramedullary hematopoiesis. The RP sinus endothelial cells provide niches for hematopoietic stem cells (Kiel et al., 2005), therefore the deviation of RP sinus endothelium specifics (reflected in their lack of Clever1 expression and IBL-9/2 staining) may be a critical factor in the defective splenic hematopoiesis. Splenic macrophages (including MARCO-positive MZ macrophages, CD169-positive metallophilic macrophages as two main MZ resident macrophages as well as F4/80-positive RP monocytes/macrophages) may also promote extramedullary hematopoiesis in stress responses (Lévesque et al., 2021). As Nkx2-3 mutant spleens have a dearth of these cells (Pabst et al., 1999; Wang et al., 2000), the resulting constitutional anemia and impaired extramedullary hematopoiesis may also be linked to these cells. Moreover, the blocked splenic hematopoiesis may be related to the formed LECs themselves as well, as recent findings indicate that the acquisition of Prox1 represses the hemogenic capacity of endothelial cells (Kazenwadel et al., 2023). Finally, recent findings have also confirmed the presence of lymphatic vasculature in both the cortical and medullary regions of human and mouse bone, and its impact on hematopoietic regeneration marrow during hematopoietic regeneration, involving their IL6-dependent LEC expansion and production of CXCL12. Subsequent recognition of CXCL12 by CXCR4-positive pericytes appears to facilitate the expansion of putative bone marrow niches for hematopoietic stem cells, which process is negatively affected by aging (Biswas et al., 2023). Detailed structural analyses performed on an extensive range of various human and murine tissues (including spleen) also revealed the progressive loss of pericytes and reduced proliferation of blood endothelial cells, accompanied by the reduced vascular density in aged individuals (Chen et al., 2021). In our study this aging-related effect was not addressed; however, the defect in upregulating extramedullary hematopoiesis (despite the presence of LYVE-1^+^/Prox1^+^/podoplanin^+^/CD31^+^ *bona fide* LECs) in Nkx2-3-mutant spleens raises the possibility of tissue-specific differences in LEC-mediated hematopoietic expansion between the bone marrow and other tissues. Clearly, further studies are needed to isolate various defined stromal subsets from Nkx2-3-deficient and wild-type mice, and compare their composition as well as gene expression characteristics.

In summary, our findings firmly establish the presence of gp38^+^/CD31^+^/Prox1^+^ LECs forming ectopic lymphatic capillaries in the spleens of mice lacking Nkx2-3, which is coupled with a phenotypic shift of RP vasculature lacking sinus-endothelium specific markers Clever1 and IBL-9/2. This shift in RP vascular patterning is associated with defective extramedullary hematopoiesis manifesting as constitutional anemia under steady-state conditions and repressed stress hematopoietic response and defective megakaryocyte expansion, together demonstrating the complex role of Nkx2-3 in shaping the vascular identity and functionality of RP.

## Data Availability

The raw data supporting the conclusions of this article will be made available by the authors, without undue reservation.
