# Supplementary figures and images for: Absence of Nkx2-3 induces ectopic lymphatic endothelial differentiation associated with impaired extramedullary stress hematopoiesis in the spleen
Source: Front Cell Dev Biol. 2023 Apr 5;11:1170389. doi: 10.3389/fcell.2023.1170389 (PMC10113473; doi:10.3389/fcell.2023.1170389)

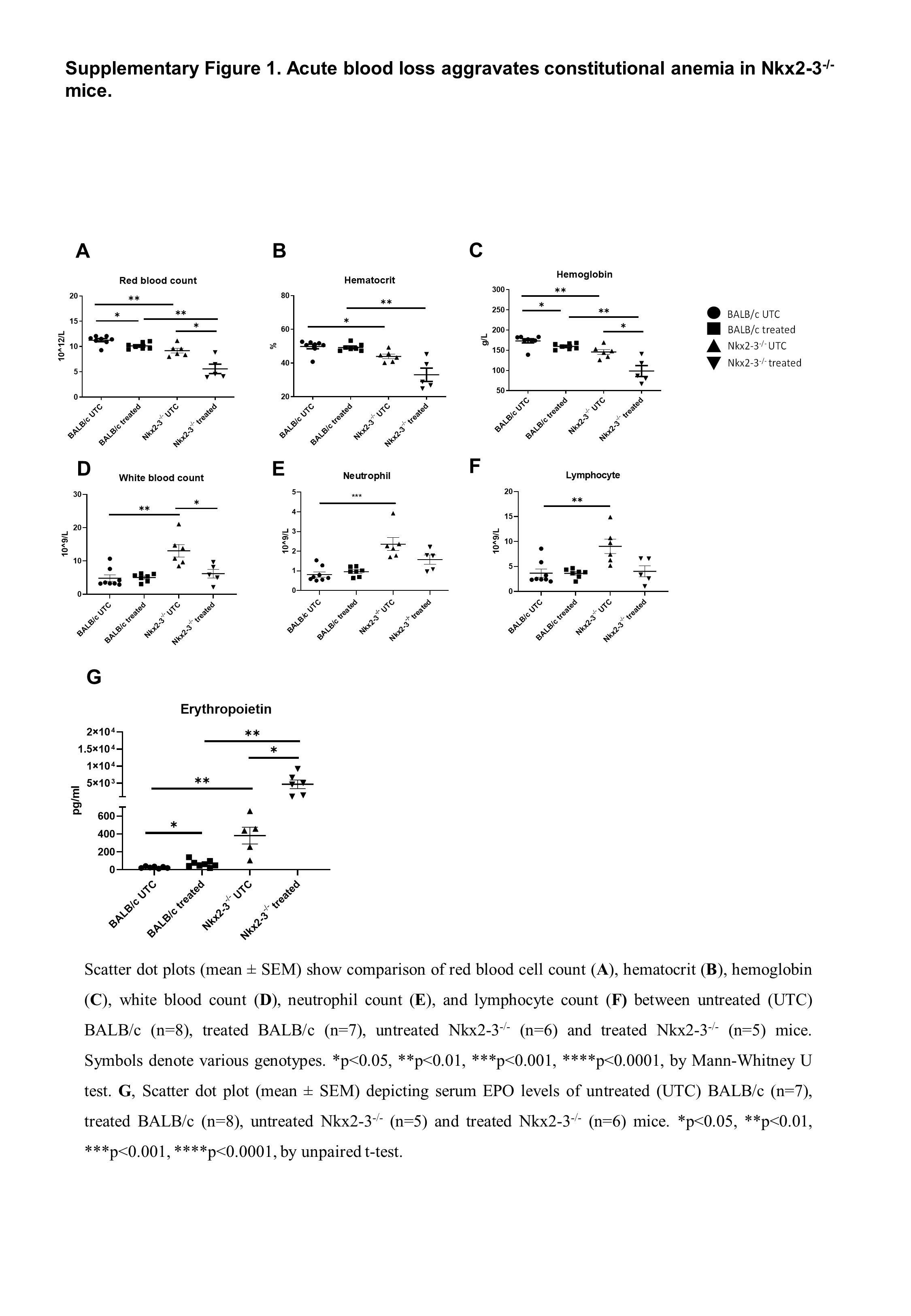

Supplement: Supplementary file 2 [file Image1.TIF]
